# Therapy of Patients with Neuroendocrine Neoplasia—Evidence-Based Approaches and New Horizons

**DOI:** 10.3390/jcm8091474

**Published:** 2019-09-16

**Authors:** Ralph A. Bundschuh, Bilel Habacha, Susanne Lütje, Markus Essler

**Affiliations:** Klinik und Poliklinik für Nuklearmedizin, Universitätsklinikum Bonn, 53127 Bonn, Germany; bilel.habacha@web.de (B.H.); Susanne.Luetje@ukbonn.de (S.L.); markus.essler@ukbonn.de (M.E.)

**Keywords:** neuroendocrine tumors, peptide receptor radionuclide therapy (PRRT), theranostics, SSTR-imaging

## Abstract

Neuroendocrine tumors (NETs) show low but increasing incidence and originate in multiple organs, including the pancreas, midgut, caecum, rectum, appendix, colon, and lungs. Due to their stunning genetic, histological, and clinical variability, diagnosis and treatment of NETs are challenging. In addition, low incidence and high variability hamper the implementation of high evidence trials. Therefore, guidelines do not cover the complexity of NETs and, frequently, treatment decisions are taken by interdisciplinary tumor conferences at comprehensive cancer centers. Treatment aims are (i) control of tumor growth, (ii) symptom control, as well as (iii) the improvement of progression-free survival (PFS) and overall survival (OS). Here, we discuss high evidence trials facilitating the achievement of these treatment aims. The majority of the evidence exists for treatment with somatostatin analogue, everolimus, peptide receptor radionuclide therapy (PRRT) with ^177^Lu-DOTATATE, sunitinib, and telotristat. Among those, PRRT is the only treatment option that has the potential to control symptoms, stop tumor growth, and to improve PFS and OS. In contrast, only a low level of evidence exists for treatment with cytotoxic drugs such as streptozotocin and doxorubicine. Finally, we discuss novel treatment options by a combination of cytotoxic drugs, ^177^Lu-DOTATATE, and tyrosine kinase inhibitors to be tested in randomized prospective trials in the future. In addition, the application of innovative isotopes, such as ^225^Ac, for PRRT is discussed.

## 1. Introduction

Neuroendocrine neoplasms (NENs) have a low incidence, but are still the most common endocrine malignancies in the western hemisphere with an increasing number of newly diagnosed cases over the past years [1]. NENs can originate from neuroendocrine cells in various organs in the body and may be located in the pancreas, midgut, caecum, rectum, appendix, colon, the lungs, or the thyroid gland. The primary location of NENs also varies by sex and race [2].

According to current World Health Organization (WHO) guidelines, NENs are split into well-differentiated NENs, defined as neuroendocrine tumors (NETs), and poorly differentiated NENs, defined as neuroendocrine carcinomas (NECs) [3]. While NECs are considered to be high-grade tumors by definition, NETs can be graded depending on their proliferation index into G1, G2, and G3 (>20%) tumors. Here, we focus on evidence-based methods in palliative treatment in NETs. Due to their high proliferation index, treatment of G3 neoplasms is mainly based on systemic antiproliferative treatments, e.g., platin-based chemotherapy [4]. However, this is beyond the scope of this paper and will not be discussed further.

In contrast to other malignancies, NETs are typically diagnosed in an advanced tumor state or incidentally in non-oncological procedures, such as appendectomy. In particular, well-differentated tumors show relatively indolent behavior with unspecific symptoms or no symptoms at all [5]. While tumor growth is relatively slow in primary tumors, metastases may grow fast, and their presence is an important prognostic factor [2]. Metastatic tumor masses along with the production of peptide hormones, such as gastrin, somatostatin, insulin, glucagon, or vaso inhibitory peptide (VIP) by tumor cells, may lead to severe symptoms and complications, such as diarrhoea, gastric ulcers, flush, diabetes, or hypoglycaemia. Low incidence along with high heterogeneity of clinical symptoms makes NETs difficult to diagnose and treat. Due to the low number of patients, controlled clinical trials are difficult to perform. Therefore, state-of-the-art treatment of NET patients should be performed at specialized and experienced centers with interdisciplinary teams, including surgeons, oncologists, endocrinologists, pathologists, as well as nuclear medicine specialists. We previously suggested a step-by-step-approach for the management of neuroendocrine tumors [6]. Examination of all NET patients by a specialized oncologist or endocrinologist is mandatory, as the clinical work-up is challenging due to heterogeneity of tumor biology and clinical symptoms. For the primary diagnosis of NETs located in the intestine or stomach, endoscopy, including endoscopic ultrasound and biopsy, is the most important method. Imaging with X-ray, computed tomography (CT), and magnetic resonance imaging (MRI), as well as somatostatin receptor (SSTR) imaging by positron-emission tomography (PET) or single-photon emission computed tomography (SPECT), are of high importance for identification of the primary tumor, as well as the detection of local and distant metastases. The recommended method of imaging, however, is dependent on the location of the primary tumor, as described in the consensus guidelines of the European Neuroendocrine Tumor Society [7]. The role of ^68^Ga-labeled SSTR-analogues in imaging of NETs is of increasing importance due to high sensitivity, specificity, and the possibility to perform a whole-body staging by a single imaging modality. As a second step after imaging, precise histological characterization is necessary [8]. Pathology is essential for therapy planning as low- and intermediate-grade (G1, G2) NETs require treatment concepts that are different to high-grade (G3) NETs or even neuroendocrine carcinomas (NECs), as already mentioned before. Therefore, assessment of proliferative activity by immunohistochemical staining of ki-67 (MIB-1) is a standard procedure in histological analysis. The proliferation rate within NETs is also correlated with prognosis and survival [9]. The third step in our approach is a comprehensive analysis of blood serum and urine parameters. Chromogranin A is the most important serum marker in NET patients to estimate tumor burden at the time of initial diagnosis and later on for monitoring of therapy response and detection of recurrence [10,11], although its use is hampered by low specificity. Another useful serum tumor marker in NETs is neuron-specific enolase (NSE), which frequently correlates with dedifferentiation and increasing aggressiveness of tumors [12]. Based on these diagnostic parameters, the most suitable therapy option should be selected. It is important to determine whether curative treatment is possible or only palliative treatment can be offered. Curative treatment can be achieved by surgery and is possible in patients with localized tumors only. Due to improved diagnostic tools, such as capsule endoscopy, and due to an increased rate of incidentally found NETs, for example, in bariatric surgery, the rate of curable NETs has been rising over the last decades. However, NETs are typically detected in late stages with metastases in the liver or mesenteric lymph nodes, which are not completely resectable. Therefore, palliative treatment plays an important role. The main aims of these concepts, at least in G1 and G2 tumors, are (i) control of symptoms, (ii) control of tumor growth and reduction of tumor mass, or (iii) prolonging progression-free survival (PFS) and overall survival (OS).

## 2. Evidence in the Treatment of Nets

Probably due to the rare incidence of NENs, but also due to long survival times, even without treatment, a limited number of studies with high evidence for medical treatment is available. Originally used for symptom control, somatostatin analogues were also evaluated for improvement of PFS and OS. While most clinical data on this topic are based on retrospective analyses or prospective open label studies, the PROMID study investigated the effect of octreotide LAR in a prospective placebo-controlled, double-blind study in 85 patients with advanced midgut NETs [13]. Treatment-naive patients were randomized either in the treatment group with 30 mg octreotide LAR monthly or in the placebo group. Median PFS was 14.3 months in the treatment group and just 6 months in the placebo group, indicating effective tumor control by octreotide LAR. Quality of life rating did not differ in the two groups. The number of serious adverse effects was not statistically significant in the treatment group as compared to the placebo group. Subsequently, in the CLARINET study, the effect of long-acting somatostatin analogue Lanreotide was analyzed in 204 patients in a randomized, double-blind, placebo-controlled study concept in grade 1 or 2 NETs, with a proliferation index (Ki-67 index) of less than 10% [14]. Only patients with documented disease progression were included. Patients in the treatment group showed statistically significant longer PFS (65.1% after 24 month) as compared to the placebo group (33.0% after 24 months). In addition, the analysis of predefined subgroups according to tumor grade (G1 vs. G2) and tumor origin (midgut, pancreas, hindgut, other) revealed significantly higher hazard ratios for disease progression in the lanreotide group than in the placebo group. However, this was not the case in the small group of hindgut NETs (*n* = 14) in which no significant difference was found. In the total cohort, quality of life was not significantly different in both groups. Moreover, no statistically significant difference in OS was observed.

Aside from the strong evidence of SSTR analogues for treatment of G1/G2 midgut tumors, many prospective studies were conducted for treatment of pancreatic NETs with the oral inhibitor of the mammalian target of rapamycin (mTOR), everolimus. In the Radiant 1 study, an open-label phase II study analyzing patients with advanced pancreatic NETs showing progression during or after chemotherapy, it was shown that daily everolimus, with or without concomitant octreotide, demonstrates antitumor activity, measured by an objective response rate and PFS [15]. Subsequently, the efficacy of everolimus compared to the placebo was investigated in the Radiant 2 study [16]. Four hundred and twenty-nine patients with low- or intermediate-grade advanced NETs were randomized either for treatment with 10 mg everolimus per day or placebo, while patients in both groups obtained octreotide LAR on a monthly basis. In the group with everolimus plus octreotide LAR, the PFS was found to be greater than in the placebo plus octreotide LAR group (median, 16.4 months vs. 11.3 months), however the difference did not reach statistical significance. In the same study, it was found that the treatment with everolimus plus octreotide LAR was associated with tumor shrinkage and stabilization, as well as reduction in biochemical markers. The effect of everolimus versus placebo in pancreatic NETs was investigated in the Radiant 3 study in 410 patients [17], while in the Radiant 4 study, 302 patients with G1 and G2 NETs of lung and gastrointestinal origin were analyzed [18]. In both studies, a prolonged PFS was found in the everolimus group compared to the placebo group, which was also shown in the subgroup analysis for NETs originating from the lung, as well as for NETs of unknown primary origin. Even in the Radiant 4 study, a trend for prolonged OS was reported (median, 23.7 months versus 16.5 months in the placebo group), although this result was not statistically significant.

The tyrosine kinase inhibitor, sunitinib (37.5 mg/d), was tested in prospective, randomized trials and compared with placebo in patients with advanced well-differentiated neuroendocrine tumors of the pancreas [19]. Median PFS was 11.4 months in the sunitinib group and 5.5 months in the placebo group. This difference was statistically significant. The objective response rate was 9.3% in the sunitinib group and 0% in the placebo group. These results indicate effective tumor control by sunitinib treatment. Moreover, a high number of serious adverse events occurred in the placebo group.

**Figure 1:**
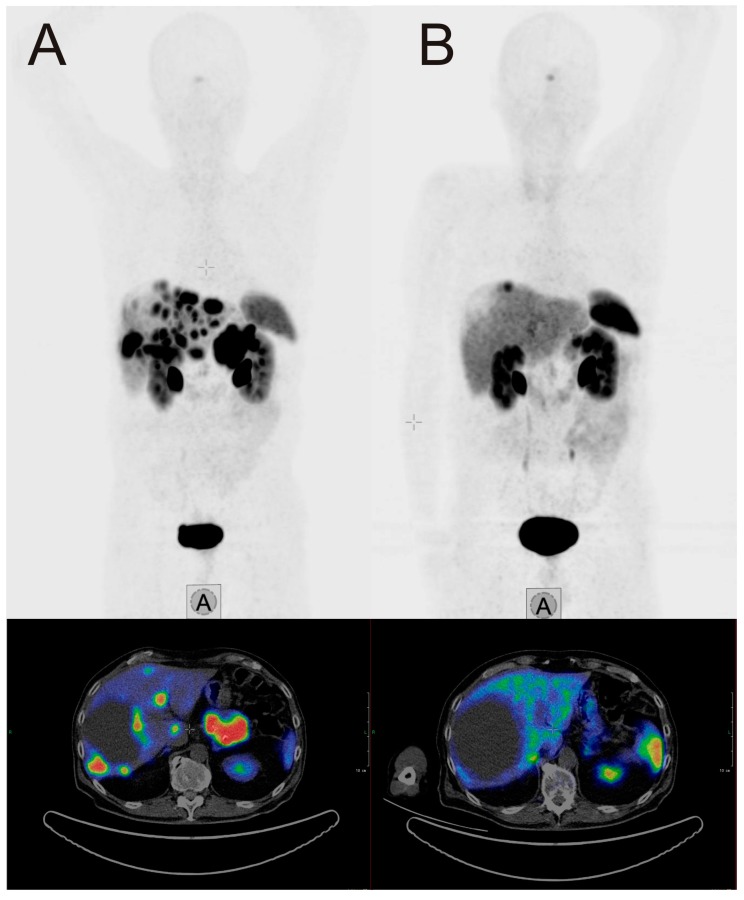
Example of a 70-year-old male patient with a pancreatic neuroendocrine tumor (NET), first diagnosed in April 2017, with an initial ki-67 index of 3%. After initial treatment with everolimus, liver metastases showed progress in November 2017. (**A**) SSTR-PET/CT at this time point. The patient was treated with four cycles ^177^Lu-DOTATATE (total dosage 29.3 GBq) between November 2017 and July 2018. Two months after the last cycle, SSTR-PET/CT (**B**) showed a very good response. Chromogranin dropped in the same interval from 1004 µg/L to 140 µg/L.

**Figure 2:**
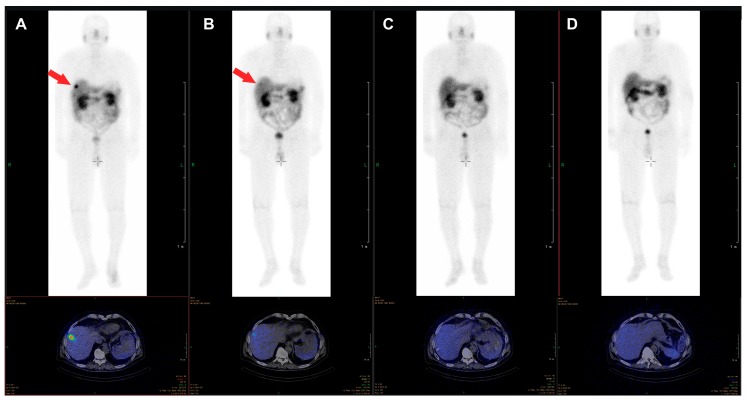
Example of a 76-year-old male patient with pancreatic NET, first diagnosed in 2012, with an initial ki-67 index of 15% who underwent four cycles of treatment with ^177^Lu-DOTATATE (cumulative dose 28.8 GBq) between October 2017 and July 2018, due to progression of bilobar liver metastases. The liver metastasis clearly visible in the post-therapy scan of the first cycle (**A**) is just barely visible in the scan of the second cycle (**B**) and not visible anymore in the scans of the third (**C**) and fourth (**D**) cycle. In the PET/CT images acquired six and twelve months after the last cycle, no tumor tissue was identified.

The β-emitter-labeled somatostatin analogue, ^177^Lu-DOTA-D-Phe-Tyr3-octreotate (DOTATATE), improves PFS, quality of live, and presumably OS. In the NETTER-1 trial, 229 patients with progressive, well-differentiated (G1 or G2) neuroendocrine tumors were randomized to be treated either with 60 mg long-acting octreotide LAR or ^177^Lu-DOTATATE and 30 mg octreotide LAR [20]. After 20 months, PFS was 62% in the group treated with ^177^Lu-DOTATATE and 30 mg Octreotide LAR compared with 11% in the Octreotide LAR group. This difference was statistically significant. The response rate was 18% in the group with radionuclide treatment vs. 3% in the group treated with Octreotide LAR. Interestingly, OS was higher in the radionuclide group too, as only 14 deaths occurred in the ^177^Lu-DOTATATE group compared to 26 in the Octreotide LAR monotherapy group. Radionuclide therapy did not cause increased rates of nephrotoxicity, however, the rate for grade 3 or 4 neutropenia, thrombocytopenia, and lymphopenia was 1%, 2%, and 9% respectively. Octreotide LAR (60 mg/d) monotherapy in contrast was not hematotoxic. In an additional analysis of the NETTER trial data, a higher quality of life was found in patients treated with ^177^Lu-DOTATATE compared to octreotide LAR monotherapy [21]. An image example of a patient with very good treatment response to ^177^Lu-DOTATATE is shown in Figure 1. The second example demonstrates a full response achieved with ^177^Lu-DOTATATE treatment. In Figure 2, images of a patient with pancreatic NETs during four cycles of treatment with a total dose of 28.8 GBq ^177^Lu-DOTATATE are presented. After the last cycle, no active tumor tissue was found in PET/CT imaging, and also in the follow-up after 6 and 12 months, no tumor localization could be identified, corresponding to a full response.

Teloristat, although being not an antiproliferative drug, was investigated in a prospective, randomized, placebo-controlled trial for control of symptoms in patients with carcinoid syndrome, not adequately controlled by somatostatin analogues [22]. Teloristat is a tryptophan hydroxylase inhibitor that slows down one of the most important steps in the serotonin biosynthesis. Due to the reduction in serotonin production in the above mentioned trial, a significant reduction of the bowel movement in the treatment group was found. An overview of these evidence-generating studies can be found in Table 1.

## 3. Treatment beyond Strong Evidence

While, as discussed above, several evidence-based treatments are available for NETs, additional treatments are in use and even included in several national guidelines (e.g., the German S2k guideline [23]), although evidence is limited or application is controversially discussed, as, for example, the role of chemotherapy in the treatment of NENs. Streptozotocin, a naturally occurring alkylating antineoplastic agent, is an approved therapy for G1/G2 NETs in many countries. However, evidence for streptozotocin treatment is based on trials that do not fulfill the criteria of modern evidence-based medicine. Moertel et al. found in 84 patients with advanced islet carcinoma that a combination of streptozotocin and 5-FU is superior to 5-FU monotherapy, as the response rate was 63% vs. 36%, respectively. However, RECIST criteria (response evaluation criteria in solid tumors) were not used. Moreover, no statistically significant improvement of PFS was found [24]. As histological analysis was not performed according to current guidelines, comparison with state-of-the-art clinical trials is difficult. The combination of streptozotocin and 5-FU was compared to the combination of streptozotocin and doxorubicin or chlorozotocin monotherapy in another randomized multicenter trial [25]. In this study, 101 patients with advanced pancreatic islet cell carcinoma were included. The combination of streptozotocin and doxorubicin had a higher response rate (69% vs. 54%) and the time to tumor progression was significantly longer (20 vs. 6.9 months). Again, response rates were determined without application of RECIST criteria. As crossover between the groups was possible, interpretation of the OS data is difficult. Interestingly, the Eastern Cooperative Oncology group performed a phase II/III trial comparing doxorubicin/5-FU and streptozotocin/5-FU in 249 patients with advanced carcinoid tumors. Patients crossed over to dacarbazine after progression under treatment with one of the two combinations. No difference after primary treatment with doxorubicin/5-FU or streptozotocin/5-FU was observed, and PFS was 4.5 vs. 5.3 months, respectively. The response rate was 15.9% vs. 16%. Remarkably, median survival was higher in the group treated with streptozotocin/5-FU (24.3 vs. 15.7 months, respectively). The response rate and PFS after crossover to dacarbazine (DTIC) were 8.2% and 11.9 months [26]. The authors concluded that the response to all three regimens was only modest, but streptozotocin/5-FU-treated patients showed the best survival. Therefore, this treatment may be applied when chemotherapy is an option in treatment of metastatic carcinoid tumors. However, it should be mentioned that some current reports, though based on retrospective analysis, showed that the combination of streptozotocin and 5-FU can have a positive effect in pancreatic NETs in terms of survival and radiological response, while having an acceptable toxicity profile [27]. In another retrospective analysis including 96 patients with advanced pancreatic NETs for the same treatment regimens, a considerable response rate was reported, and treatment was associated with extended time to progress, especially in the case of a proliferation index equal to or lower than 15% [28].

The situation is comparable for interferon alpha (IFN). Kölby et al. tested the effect of IFN on survival of patients with disseminated midgut carcinoid tumors [29]. In a prospective, randomized trial with 68 patients treated with octreotide or a combination of IFN and octreotide, no significant difference in survival after five years was observed. However, the combination group had a reduced risk of tumor progression during follow-up. Remarkably, PFS was not determined. Therefore, the results are difficult to compare with trials having appropriate endpoints. In another study, IFN plus octreotide was compared with bevacizumab plus depot octreotide in 427 patients with advanced carcinoid tumors in a prospective, randomized phase III trial [30]. No significant differences in PFS (16.6 vs. 15.4 months) or adverse effects were found in this study, indicating that both combinations have similar anti-tumor effects. On the other hand, this trial is difficult to interpret as, according to the authors’ conclusions, the value of IFN monotherapy is not clear.

Additionally, in case of liver metastases dominating the disease, patients may benefit from additional local treatment with selective internal radioembolization in parallel with everolimus and pasireotide [31].

## 4. Conclusions and Outlook

Biotherapy with somatostatin analogues, the mTOR inhibitor everolimus, the VEGF inhibitor sunitinib, and ^177^Lu-DOTATATE radionuclide therapy has emerged as evidence-based treatment of advanced G1/G2 neuroendocrine tumors prolonging PFS and/or OS. Additionally, the tryptophan hydroxylase inhibitor teloristat provides evidence-based treatment of symptoms of the carcinoid syndrome. In addition to evidence-based therapies, current guidelines include chemotherapy and interferon. While there is a large body of papers about chemotherapy and interferon in NET patients, there are no phase III trials according to the current rules for evidence-generating trials. Low patient numbers along with high biological heterogeneity of NETs cause this lack of evidence. Most chemotherapeutic drugs for treatment of NETs are not protected by patents. Thus, new prospective trials testing, for example, streptozotocin are unlikely to be performed.

So far, it is difficult to compare the value of most of these treatments directly as no head to head trials exist. Therefore, network meta-analysis was used to compare multiple treatments in advanced well-differentiated (G1/G2) NETs [32]. In this evaluation, 21 papers on about 15 randomized controlled trials with 2922 patients receiving 11 treatments were included. The authors found peptide receptor radionuclide therapy (PRRT) plus octreotide to be the therapy with the highest improvement of PFS, while having acceptable adverse events comparing efficacy and side effects of the different treatments. Somatostatin analogues plus bevacizumab and IFN plus somatostatin analogues ranked second and third. In our opinion, it is not clear why bevacizumab ranks higher compared to sunitinib in this analysis as sunitinib treatment clearly improves PFS in advanced NETs. Regardless of this issue, tyrosine kinase inhibitors blocking angiogenesis may play an important role in future treatment of NETs.

Aside from the lack of evidence for several treatment options, another problem is the decision for the best treatment sequence. As there are several evidence-based treatment options available, there are different sequences to apply them. As there is no clear evidence-based answer for this question, in our opinion, it is important to make these decisions within an interdisciplinary team including surgeons, oncologists, endocrinologists, pathologists, and nuclear medicine specialists. Therefore, treatment of NET patients should be performed at specialized and experienced centers. Such a step-by-step approach for the management of neuroendocrine tumors was suggested by our group before [6] as well as by others [33].

We propose that in the future, therapy of NETs will be guided by analysis of the molecular patterns in addition to conventional histopathology and tissue of origin. It may be possible to conduct trials with molecular features as the inclusion criteria instead of location of the primary tumor. Thereby, a higher number of patients that are eligible for such trials may be available, and it may be possible to overcome the lack of evidence. Several genetic markers have been reported to be of interest in NENs, although no systematical analyses of their prognostic value have been made. Vijayvergia et al. described TP53, BRAF, DAXX, and ATRX as potential genes of the most prevalent mutations in poorly differentiated NECs [34]. Several other studies have shown that ATRX, DAXX, MEN1, TP53, ATM, and mTOR pathway related genes are often mutated in pancreatic NETs in a somatic fashion. Chromosome instability and reduced survival have been associated with the ATRX and DAXX mutations in pancreatic NETs as well [35]. One of the most well-known mutations leads to the multiple endocrine neoplasia syndrome, which has poor prognosis. The pancreatic and thyme NETs are the deadliest of the MEN1 lesions and they often develop into metastatic disease. Facial angiofibromas, collagenomas, lipomas, and meningiomas have been shown to occur frequently in MEN1 patients compared to the other non-endocrine tumors [35]. Interestingly, another gene that belongs to the P13K/mTor pathway involved in inhibiting P13K, namely PTEN, was identified as a periodically mutated tumor suppressor, which confirms previous studies on the recurrent mutation of MEN1 [36].

As well as histopathological features, radiomics features may play a role in treatment decisions in the future. As reported recently, analysis of textural features as a surrogate marker for tumor heterogeneity in pre-therapeutic SSTR-PET in 141 patients can predict post-therapeutic time-to-progress and OS in treatment with ^177^Lu-labeld SSTR-analogues [37]. Similar results were found in a collective with 31 pancreatic NETs [38]. However, the high number of histopathological markers, serum markers, as well as radiomic features often investigated in different studies makes it very difficult to use the features for treatment decisions. In the future artificial intelligence may be the key technology to combine all these parameters to end up with a probability for which treatment at which time point will be the optimal decision for each patient. This would be the next step for individualized tumor therapy in patients suffering from NETs.

Aside from which chosen treatment can be performed best, radionuclide therapy with ^177^Lu-labeled SSTR analogues will also play a more important role in treatment of NETs in the future. PRRT is the only treatment for advanced G1/G2 NETs that improves OS according to a prospective randomized trial, improves PFS, reduces tumor load, and helps to control symptoms. Currently, trials are performed to compare the effectivity of PRRT with everolimus. Everolimus is the standard therapy for advanced NETs after progression under treatment with somatostatin analogues. The COMPETE (ClinicalTrials.gov identifier NCT03049189) trial is an international prospective, randomized phase III trial comparing the efficacy and safety of the radiolabeled somatostatin analogue ^177^Lu-Edotreotide with everolimus. The superior of both therapies will be used first after progression of NETs under treatment with somatostatin analogues.

The effect of PRRT may be enhanced by use of different isotopes, such as the high-energy beta emitter yttrium-90 (^90^Y) or the alpha emitter actinium-225 (^225^Ac) instead of ^177^Lu. Conjugates of ^225^Ac isotopes were tested in preclinical models. Successful treatment cases were reported anecdotally and in retrospective studies, but not in prospective clinical trials [39], while SSTR analogues labeled with ^90^Y have already been applied in humans [40]. As radiation from ^90^Y or ^225^Ac has different physical properties (energy, range, and linear energy transfer) compared to ^177^Lu, these isotopes may have higher anti-tumor activity, but it is possible that they also cause more severe side effects. Therefore, the therapeutic window of SSTR analogues labeled with ^90^Y or ^225^Ac has to be determined in prospective trials. It is also a matter of debate how many cycles of ^177^Lu-DOTATOC can be performed safely in case of recurrence. A retrospective analysis showed recently that up to 13 cycles of ^177^Lu-DOTATOC may be performed with an acceptable rate of toxic side effects [41]. Therefore, prospective trials to address this issue may be valuable too. Combination with other types of therapy may enhance the effect of PRRT. Interestingly, somatostatin analogues potentially enhance the effect of ^177^Lu-DOTATOC. The NETTER trial did not include a treatment arm with PRRT only. Therefore, the value of PRRT compared to PRRT plus Octreotide LAR was not studied in this trial. A retrospective analysis analyzing a collective of 168 patients with advanced NETs treated with PRRT or PRRT plus octreotide showed that in the combination treatment group, PFS was significantly higher compared to PRRT only [42]. These results have to be confirmed in prospective trials and the underlying molecular mechanisms have to be studied. Furthermore, combinations of chemotherapy with PRRT were suggested [43]. These combinations were promising in small phase I/II trials and in retrospective settings, but there are still no prospective data from controlled trials available.

Although it has been reported that PRRT improves quality of life of patients with pancreatic NETs as well as midgut NETs in prospective and retrospective studies [21,44], the long-term effect of isotopes is controversial. In some retrospective trials, an increased rate of acute myelogenous leukemia has been reported [45]. The prospective data from the NETTER-1 trial will have to be evaluated in the future for the rate of post-therapeutic leukemia to clarify this issue.

In conclusion, evidence-based treatments of advanced or intermediate differentiated NETs should be preferred. The value of somatostatin analogues, everolimus, sunitinib, telotristat, and ^177^Lu-DOTATOC is well documented by evidence-generating trials. The sequence of application of these and less understood treatments needs to be determined by interdisciplinary tumor boards, based on the individual situation of the patients and published guidelines. In future, molecular analysis and radiomics features may be helpful to improve treatment of patients with G1/G2 NETs.

## Figures and Tables

**Table 1:** List of the evidence-generating studies for the treatment of NETs.

Trial	Drug	Indication	Result	Year	Citation
PROMID	Octreotide LAR vs. placebo	Treatment-naive, locally inoperable, or metastatic midgut NETs	Increased PSF	2009	[13]
Radiant 1	Everolimus +/− octreotide LAR	Advanced pancreatic NETs after chemotherapy	Increased PFS and response rate	2010	[15]
Radiant 2	Everolimus + octreotide LAR vs. placebo + octreotide LAR	Advanced G1 and G2 NETs with carcinoid syndrome	Increased PFS	2011	[16]
Radiant 3	Everolimus	Progressive pancreatic NETs (G1 and G2)	Increased PFS	2011	[17]
	Sunitinib vs. placebo	Pancreatic NETs	Increased PFS, OS and response rate	2011	[19]
CLARINET	Lanreotide vs. placebo	Advanced grade 1 and 2 non-functioning enteropancreatic NETs	Increased PFS, unchanged OS	2014	[14]
Radiant 4	Everolimus	Progressive NETs from lung or GU (G1 or G2)	Increased PFS	2016	[18]
NETTER-1	^177^Lu-DOTATATE vs. high-dose LAR	Midgut Neuroendocrine Tumors	Increased PFS	2017	[20]
TELESTAR	Teloristat	Carcinoid syndrome not controlled by SA	Reduced bowel motion	2016	[22]

The grey background indicates, that the drug investigated in this study was not analyzed for antiproliferative effects but for symptom control.
